# Racial/ethnic differences in acute and longer-term posttraumatic symptoms following traumatic injury or illness

**DOI:** 10.1017/S0033291722002112

**Published:** 2023-08

**Authors:** Mario Cruz-Gonzalez, Margarita Alegría, Patrick A. Palmieri, David A. Spain, M. Rose Barlow, Lisa Shieh, Mallory Williams, Pranathi Srirangam, Eve B. Carlson

**Affiliations:** 1 Disparities Research Unit, Department of Medicine, Massachusetts General Hospital, Boston, MA, USA; 2 Department of Medicine, Harvard Medical School, Boston, MA, USA; 3 Department of Psychiatry, Harvard Medical School, Boston, MA, USA; 4 Traumatic Stress Center, Department of Psychiatry, Summa Health, Akron, OH, USA; 5 Department of Surgery, Stanford University School of Medicine, Stanford, CA, USA; 6 National Center for PTSD, Dissemination and Training Division, VA Palo Alto Health Care System, U.S. Department of Veterans Affairs, Menlo Park, CA, USA; 7 Department of Medicine, Stanford University School of Medicine, Stanford, CA, USA; 8 Department of Surgery, Howard University College of Medicine, Washington, DC, USA; 9 Center of Excellence in Trauma and Violence Prevention, Howard University College of Medicine, Washington, DC, USA; 10 Barnard College of Columbia University, New York, NY, USA; 11 Department of Psychiatry and Behavioral Sciences, Stanford University School of Medicine, Stanford, CA, USA

**Keywords:** Emergency care, pre-, peri-, and post-trauma risk factors, posttraumatic symptoms, race and ethnicity, traumatic injury or illness

## Abstract

**Background:**

Racial/ethnic differences in mental health outcomes after a traumatic event have been reported. Less is known about factors that explain these differences. We examined whether pre-, peri-, and post-trauma risk factors explained racial/ethnic differences in acute and longer-term posttraumatic stress disorder (PTSD), depression, and anxiety symptoms in patients hospitalized following traumatic injury or illness.

**Methods:**

PTSD, depression, and anxiety symptoms were assessed during hospitalization and 2 and 6 months later among 1310 adult patients (6.95% Asian, 14.96% Latinx, 23.66% Black, 4.58% multiracial, and 49.85% White). Individual growth curve models examined racial/ethnic differences in PTSD, depression, and anxiety symptoms at each time point and in their rate of change over time, and whether pre-, peri-, and post-trauma risk factors explained these differences.

**Results:**

Latinx, Black, and multiracial patients had higher acute PTSD symptoms than White patients, which remained higher 2 and 6 months post-hospitalization for Black and multiracial patients. PTSD symptoms were also found to improve faster among Latinx than White patients. Risk factors accounted for most racial/ethnic differences, although Latinx patients showed lower 6-month PTSD symptoms and Black patients lower acute and 2-month depression and anxiety symptoms after accounting for risk factors. Everyday discrimination, financial stress, past mental health problems, and social constraints were related to these differences.

**Conclusion:**

Racial/ethnic differences in risk factors explained most differences in acute and longer-term PTSD, depression, and anxiety symptoms. Understanding how these risk factors relate to posttraumatic symptoms could help reduce disparities by facilitating early identification of patients at risk for mental health problems.

Each year, millions of patients in the USA are hospitalized following traumatic injury or illness (Rui, Kang, & Albert, 2013), many of whom are at risk for posttraumatic stress disorder (PTSD), depression, and anxiety (Carlson, Palmieri, & Spain, 2017; Grant, Beck, Marques, Palyo, & Clapp, 2008). A recent study found that 25.1% and 11.5% of patients receiving emergency care after a motor vehicle crash (the most common trauma in industrialized countries) met criteria for 3-month PTSD and depression, respectively (Ziobrowski et al., 2021). Risk for posttraumatic symptoms has also been found to differ by racial and ethnic groups. Prior studies of hospitalized injury survivors showed that Latinx and Black patients had significantly elevated risk for developing early and 12-month PTSD symptoms relative to White patients (Santos et al., 2008; Zatzick et al., 2007). Another USA population-based study also found that Black patients hospitalized following an acute injury had significantly higher risk of early PTSD symptoms than White patients (Stephens et al., 2010). In contrast, a recent study found no racial/ethnic differences in PTSD symptoms within 3-months after trauma, but lower posttraumatic anxiety and depression symptoms in Black patients relative to White patients, which were accounted for by prior trauma exposure and childhood emotional abuse (Harnett et al., 2022). There is limited research examining whether additional risk factors prior to, at the time of, and post-injury or illness might explain racial/ethnic differences in acute and longer-term posttraumatic symptoms.

Prior research has suggested that racial/ethnic differences in posttraumatic stress may result from differences in sociodemographic and clinical factors, the type and number of traumatic exposures, and support from family and friends (Alegría et al., 2013). Social support, including negative social reactions in response to trauma disclosure and social constraints that lead the trauma survivor to feel unsupported or misunderstood, has also been identified as one of the most robust predictors of posttraumatic stress (Zalta et al., 2021). A prior study found that variations in social support explained differences in PTSD between Black and White inner-city women in the USA (Gaffey et al., 2019). Racial/ethnic identity may also be a proxy for experiences of minoritized groups that confer greater risk for mental health problems (Holzer & Copeland, 2013). For example, US racial/ethnic minority populations are more likely to live in high-stress environments (Jackson, Knight, & Rafferty, 2010), and pre-trauma life stress has been found to be strongly associated with posttraumatic stress (Carlson et al., 2016). Racial/ethnic disparities in posttraumatic stress could also contribute to socioeconomic disadvantages and cycles of violence, so that responses to trauma can lead to additional trauma exposure (Eitle & Turner, 2002).

According to the sociocultural framework for mental health disparities (Alegría, Pescosolido, Williams, & Canino, 2011), competing needs and discrimination may also explain racial/ethnic differences in trauma responses. Competing needs are basic social and economic resources; when unmet, they can impair recovery after trauma by creating barriers to addressing mental health needs – including transportation, financial, and information barriers (Davis, Ressler, Schwartz, Stephens, & Bradley, 2008). Discrimination in the form of major, acute experiences and daily, low level microaggressions is also related to racial/ethnic disparities in mental health (Torres & Taknint, 2015; Williams & Mohammed, 2009). Discrimination in criminal justice, political, and immigration contexts may also lead to negative expectations and feelings of hopelessness (Utsey & Ponterotto, 1996). Recent evidence demonstrates that racial discrimination increased the risk of developing future PTSD symptoms following traumatic injury among Black patients (Bird et al., 2021).

Using a sample of patients hospitalized after emergency care following severe injury or illness, we examined whether there were racial/ethnic differences in acute and longer-term PTSD, depression, and anxiety symptoms, and whether pre-, peri-, and post-trauma risk factors explained such differences. Severely injured or ill patients were included since the ICD-11 and DSM-5 diagnostic systems both specify injury and life-threatening illness as potential traumatic stressors. Using a theoretical framework delineating the risk factors associated with posttraumatic symptoms (Carlson, Dalenberg, & Muhtadie, 2008) in combination with the sociocultural framework for mental health disparities (Alegría et al., 2011), we examined how pre-, peri-, and post-trauma risk factors interacted with race/ethnicity to reflect mechanisms of action that might confer vulnerability or resilience to traumatic stress, exacerbate distress in response to trauma, or foster rapid recovery after exposure. We hypothesized that Latinx and Black patients would report higher levels of PTSD, depression, and anxiety symptoms than White patients, and that these differences would be accounted for by pre-, peri-, and post-trauma risk factors that could influence trauma response.

## Method

### Participants and procedure

Participants were 1320 adult patients (18–89 years old) admitted after emergency care following severe injury (*n* = 661) or illness (*n* = 658) at hospitals in Palo Alto, CA, Akron, OH, and Baltimore, MD. Injuries included motor vehicle crash, being hit by a car, physical assault, falling, work injury, knife or gunshot, sport injuries, burns, and animal bites. Illnesses included heart, breathing, gastrointestinal, or liver problems; sepsis or bad infections; brain, nerves, vision, or speech problems; problems with blood sugar and diabetes; sickle cell disease; kidney problems; and cancer. Using self-reported information, patients were coded into mutually exclusive groups as ‘Asian, Native Hawaiian, or Other Pacific Islander’ (‘Asian’; *n* = 91), ‘Hispanic or Latino’ (‘Latinx’; *n* = 196), ‘non-Hispanic Black’ (‘Black’; *n* = 310), ‘multiracial’ (*n* = 60), ‘non-Hispanic White’ (‘White’; *n* = 654), ‘American Indian or Alaska Native’ (*n* = 7), and ‘other-race’ (*n* = 2). Due to small sample size, American Indian or Alaska Native and other-race patients (*n* = 9) were excluded, and so was the only White patient reporting their gender as ‘other’. Thus, our final analytical sample included 1310 patients.

Patients were identified via electronic records and approached in their hospital rooms soon after admission (mean = 4 days, median = 3 days, mode = 2 days) between June 2018, and February 2021 (time 1). Eligible patients had cognitive capacity to answer study questions and were able to provide contact information for follow-up assessments. Patients admitted for psychiatric treatment were excluded. After providing informed consent, patients completed all study measures at time 1 using tablet computers, paper questionnaires, or orally. Bilingual staff recruited patients who spoke or read English (*n* = 1216), Spanish (*n* = 87; all but one Latinx patients), or Mandarin (*n* = 7; all Asian patients). Repeated measures of posttraumatic symptoms were collected in two follow-up assessments at 2 and 6 months post-admission (times 2 and 3). Trauma responses did not differ at any time point between patients admitted after injury or illness. We also found no differences in trauma responses between Latinx patients who completed their assessments in Spanish or English, between Asian patients who completed their assessments in Mandarin or English, or between patients admitted before or during the COVID-19 pandemic (i.e. before *v.* on or after March 2020). There were racial/ethnic differences in follow-up response rates [χ^2^(12) = 109.68, *p* < 0.01]. Analyses of missing data showed that within racial/ethnic groups, patients who completed all follow-up assessments were statistically similar to patients who missed at least one follow-up assessment, which made plausible assuming that data were missing at random. Thus, we applied multiple imputation methods following a prior study of repeated patient-reported measures with high rates of missing data (Biering, Hjollund, & Frydenberg, 2015; see details in online Supplementary material).

### Study measures

Good internal consistency (Cronbach's *α*) was observed for all measures included in the present study across all racial and ethnic groups (a complete breakdown is presented in online Supplementary Table S1).

#### Outcomes: PTSD, depression, and anxiety symptoms at times 1–3

Symptoms were rated on a six-point scale from 0, ‘none of the time/not at all’, to 5, ‘all or most of the time/four or more times a day’. The time reference was *since the time of hospitalization* at time 1 and *in the past week* at times 2 and 3. A total symptom severity score was derived by summing all items, with higher scores representing worse symptoms. At time 1, measures of depression and anxiety were shortened to reduce burden on acutely injured or ill patients based on full measures collected among the first 518 patients. These shortened versions were used at all time points in the current analyses. PTSD symptoms were evaluated using the Screen for Posttraumatic Stress Symptoms (Carlson, 2001), a 20-item self-report assessing PTSD symptoms according to DSM-5 criteria (range = 0–100). Depression symptoms were assessed using six of the nine items of the Patient Health Questionnaire-9 (PHQ-9) (Kroenke & Spitzer, 2002), a self-report assessing symptoms of DSM-IV depressive disorders (range = 0–30, *r* = 0.97 with full PHQ-9). Anxiety symptoms were assessed with two of the seven items of the Generalized Anxiety Disorder-7 (GAD-7) (Spitzer, Kroenke, Williams, & Löwe, 2006) – a self-report assessing GAD symptoms according to DSM-IV diagnostic criteria – plus three novel items assessing anxiety independent of a diagnosis and using updated language developed for the present study (range = 0–25, *r* = 0.98 with full GAD-7 plus three novel items).

#### Pre-trauma risk factors from time 1

Sociodemographic data included gender (female, male), age (18–89 years old), marital status (never married, married/cohabitating, separated/divorced/widowed, other), years of education (0–11, 12, 13–15, 16+), and household income (<$35 K, $35 K to <$75 K, $75 K+). Two early-life risk factors highly predictive of mental health outcomes (Carlson et al., 2016) were assessed using measures of childhood home life (*How was your home life growing up*?, 1 ‘unhappy’ to 5 ‘very happy’) and caretaker dysfunction (sum of affirmative responses about whether any caretaker ever stayed in a hospital for emotional or psychiatric reasons, attempted or completed suicide, abused drugs or alcohol, or was arrested). Two items for number of lifetime sudden, terrible events, and how many of these events were upsetting for a month or longer assessed past trauma exposure. Both items were censored at the 98th percentile (50 and 25 events, respectively) to reduce the effect of extreme outliers, and averaged to create a summary score (range = 0–37.5).

The nine-item Everyday Discrimination Scale (EDS) was used to capture everyday experiences with unfair treatment that are chronic or episodic but generally minor (0 ‘never’ to 5 ‘almost every day’, range = 0–45) (Kim, Sellbom, & Ford, 2014; Williams & Mohammed, 2009). Extreme discrimination was assessed using the Race-Related Events Scale (RRES), a brief screening of lifetime exposure to stressful and potentially traumatizing experiences due to race/ethnicity, gender, or religion (Waelde et al., 2010). A count of 12 RRES items not overlapping with the EDS were included (e.g. verbal conflict, physical fight/assault, being chased). Financial stress was measured with three items from past epidemiological research (Alegria et al., 2004) about whether patients would say they have enough money to meet their needs (0 ‘more than I need’ to 2 ‘not enough’), whether it was hard to pay their monthly bills (0 ‘not at all hard’ to 3 ‘very hard’), and how often in the past 12 months they did not have enough money to buy food (0 ‘never’ and 3 ‘often’). Total scores were computed summing all items (range = 0–8). Past mental health problems were measured with one item asking how much of the time in the past had feeling anxious, down, or depressed kept the patient from enjoying life (0 ‘not at all’ to 5 ‘all or almost all the time’).

#### Peri-trauma risk factors from time 1

Two items assessing patients' perception of how terrible and out of control the event that brought them to the hospital seemed (0 ‘not at all’ to 4 ‘a lot’) were used to measure subjective trauma severity. Total scores were calculated as the sum of both items (range = 0–8).

#### Post-trauma risk factors from time 1

Expected social support was assessed with six items from the Medical Outcomes Study Social Support Survey, a self-report of recent thinking about four dimensions of social support: tangible, affectionate, emotional/informational, and positive social interaction (Sherbourne & Stewart, 1991). Patients were asked how much support they think they would get – if needed – after leaving the hospital (0 ‘none of the time’ to 5 ‘all or most of the time’, range = 0–30). Social constraints were assessed with seven items from the Leopore Social Constrains Scale, a self-report of perceived friends and family constraining behaviors on expressing trauma-related thoughts and feelings (e.g. friends or family act uncomfortable when you talk about the event that brought you to the hospital; 0 ‘none of the time’ to 5 ‘all or most of the time’, range = 0–35) (Lepore & Ituarte, 1999).

### Data analysis plan

We first described differences in risk factors at time 1 between racial/ethnic minority and White patients. Using individual growth curve models, we then examined racial/ethnic differences in PTSD, depression, and anxiety symptoms at the time of hospitalization (time 1) and 2 and 6 months post-admission (times 2 and 3). These models also examined racial/ethnic differences in the rate of PTSD, depression, and anxiety symptoms change over time. Symptoms at times 1–3 were separately modeled as the dependent variable. Individual growth curve models allowed us to (1) account for the correlation between repeated measures from the same patient, (2) account for racial/ethnic differences in responses at each time point, and (3) examine how responses changed over time within patients and how that change was related to a patient's race/ethnicity. The final equation was as follows:1
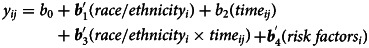
where *y*_*ij*_ represents patient *i*'s symptoms at time *j*, *time*_*ij*_ represents follow-up time (coded 0, 2, and 6 at times 1, 2, and 3, respectively), *race*/*ethnicity*_*i*_ indicates patient *i*'s racial/ethnic identity, and *risk factors*_*i*_ are a vector of pre-, peri-, and post-trauma risk factors at time 1. The parameter *b*_0_ represents symptoms at time 1 for the referent group (White patients), and the vector ***b***_1_ measures differences in symptoms at time 1 between racial/ethnic minority and White patients. The parameter *b*_2_ measures symptoms change over time for White patients, and the vector ***b***_3_ measures differences in the rate of change between racial/ethnic minority and White patients. We estimated two sets of models, with and without adjustment for risk factors. In post-hoc analyses, we expanded the model from Equation (1) to examine whether risk factors at time 1 partially explained racial/ethnic differences in both outcome scores at each time point and outcome score change over time. To avoid model overspecification, we estimated separate models considering the role of each risk factor in explaining racial/ethnic differences. Analyses were conducted using the Stata software version 15 (StataCorp, 2017) and the RStudio software version 1.4.1717 (RStudio Team, 2021; see online Supplementary Material for additional details).

## Results

### Racial/ethnic differences in risk factors

There was racial/ethnic variation for most pre-, peri-, and post-trauma risk factors at time 1, and specific patterns varied across risk variables (Table 1). Compared to White patients, Asian patients were the most likely to report being married or cohabitating, having the most education and income, and the lowest levels of caretaker dysfunction and financial stress. Latinx patients were the most likely to report not having a high school degree, the lowest levels of everyday discrimination, and the highest levels of subjective trauma severity and social constraints. Black patients were the most likely to report never being married and not being in the highest household income category; they also had the second highest levels of everyday discrimination, extreme discrimination, and financial stress, and the second lowest levels of expected social support. Multiracial patients were the most likely to report not having a college degree, being in the lowest household income category, being unhappy about their childhood home life, having the highest levels of caretaker dysfunction, everyday discrimination, extreme discrimination, and financial stress, and the lowest levels of expected social support.

**Table 1.** Descriptive statistics of risk factors at the time of hospitalization (time 1) by race and ethnicity

Risk factor, mean (s.e.)	Asian	Latinx	Black	Multiracial	Referent group: White	*F* value;^a^ *p* value
	(*N* = 91)	(*N* = 196)	(*N* = 310)	(*N* = 60)	(*N* = 653)	
*Pre-trauma risk factors*						
Demographics						
Gender						
Female	51.6% (5.3%)	39.8% (3.5%)	47.4% (2.8%)	45.0% (6.5%)	49.3% (2.0%)	1.6; *p* = 0.17
Male	48.4% (5.3%)	60.2% (3.5%)	52.6% (2.8%)	55.0% (6.5%)	50.7% (2.0%)	
Marital status						
Never married	26.6% (4.7%)	28.2% (3.3%)	44.6% (2.9%)	35.5% (6.3%)	22.7% (1.7%)	11.4; *p* < 0.01
Married/cohabitating	64.3% (5.1%)	46.5% (3.6%)	30.9% (2.7%)	36.6% (6.4%)	51.2% (2.0%)	13.5; *p* < 0.01
Separated/divorced/widowed	7.9% (2.9%)	24.7% (3.1%)	21.6% (2.4%)	24.4% (5.7%)	24.8% (1.7%)	6.8; *p* < 0.01
Other	1.2% (1.2%)	0.6% (0.6%)	2.9% (1.0%)	3.5% (2.4%)	1.3% (0.4%)	1.3; *p* = 0.28
Education level						
0–11 years	7.7% (2.8%)	42.3% (3.5%)	13.2% (1.9%)	6.7% (3.2%)	6.1% (0.9%)	25.9; *p* < 0.01
12 years (high school)	12.1% (3.4%)	20.9% (2.9%)	32.9% (2.7%)	23.3% (5.5%)	27.9% (1.8%)	6.8; *p* < 0.01
13–15 years	20.9% (4.3%)	30.1% (3.3%)	40.0% (2.8%)	65.0% (6.2%)	32.9% (1.8%)	10.2; *p* < 0.01
16+ years	59.3% (5.2%)	6.6% (1.8%)	13.9% (2.0%)	5.0% (2.8%)	33.1% (1.8%)	49.2; *p* < 0.01
Household income						
Less than $35 K	23.7% (5.1%)	59.1% (4.7%)	70.0% (2.9%)	71.0% (6.6%)	35.8% (2.0%)	31.0; *p* < 0.01
$35 K to less than $75 K	12.5% (4.3%)	25.3% (3.8%)	21.8% (2.6%)	14.3% (5.2%)	26.4% (1.9%)	3.2; *p* = 0.01
$75 K and more	63.8% (5.7%)	15.6% (3.2%)	8.2% (1.8%)	14.6% (5.1%)	37.8% (2.0%)	44.1; *p* < 0.01
Early life						
Caretaker dysfunction	0.28 (0.08)	0.71 (0.08)	0.71 (0.06)	1.18 (0.18)	0.69 (0.04)	8.7; *p* < 0.01
Past trauma exposure						
Lifetime sudden/terrible events^b^	4.93 (1.01)	4.38 (0.53)	5.13 (0.44)	9.18 (1.83)	5.25 (0.31)	1.8; *p* = 0.13
Perceived discrimination						
Everyday discrimination	6.42 (0.84)	5.51 (0.66)	9.66 (0.56)	11.30 (1.40)	7.26 (0.32)	8.4; *p* < 0.01
Extreme discrimination	1.34 (0.26)	2.11 (0.24)	2.55 (0.19)	3.36 (0.53)	1.26 (0.10)	13.6; *p* < 0.01
Financial stress	2.26 (0.20)	4.02 (0.18)	4.45 (0.12)	4.59 (0.29)	2.98 (0.09)	36.8; *p* < 0.01
Past mental health problems	1.15 (0.11)	1.08 (0.07)	1.14 (0.07)	1.28 (0.16)	1.10 (0.04)	0.4; *p* = 0.80
*Peri-trauma factors*						
Subjective trauma severity^c^	3.89 (0.22)	4.36 (0.13)	3.82 (0.12)	4.07 (0.29)	4.02 (0.08)	2.4; *p* = 0.047
*Post-trauma factors*						
Expected social support	24.27 (0.89)	22.79 (0.67)	21.73 (0.53)	20.07 (1.24)	23.45 (0.34)	3.8; *p* < 0.01
Social constraints	3.99 (0.62)	6.10 (0.46)	5.21 (0.40)	5.63 (0.92)	3.24 (0.22)	11.2; *p* < 0.01

a *F* value of omnibus test comparing non-Latinx White patients and all other racial/ethnic groups.

b Number of lifetime sudden/terrible events were Winsorized to the 98th percentile to reduce the effect of extreme outliers.

c Subjective trauma severity was assessed using two items about how ‘terrible’ and ‘out of control’ the event that brought participants to the hospital seemed (0 ‘not at all’ and 4 ‘a lot’).

### Racial/ethnic differences in PTSD, depression, and anxiety symptoms

**Fig. 1.**
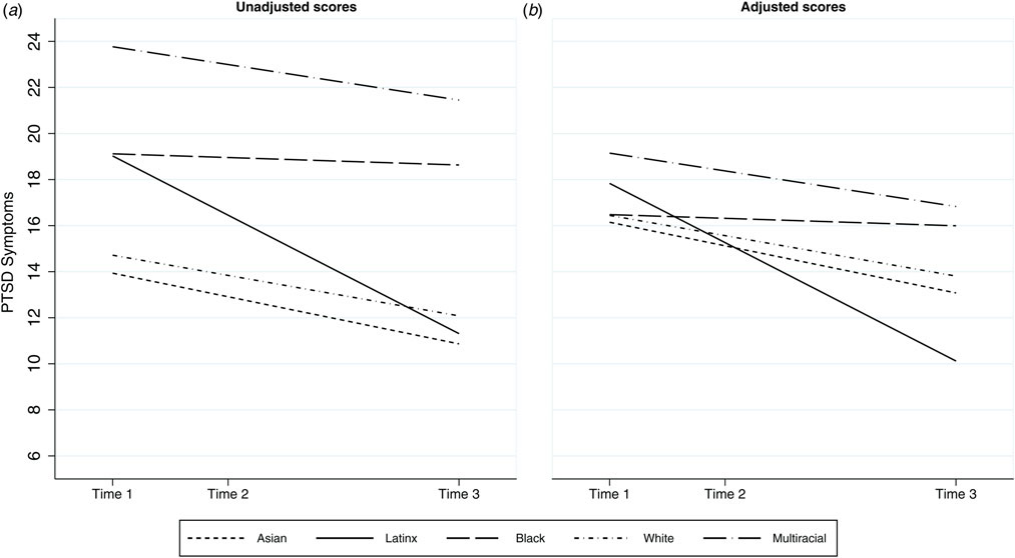
Unadjusted and adjusted mean PTSD scores at the time of hospitalization (time 1), 2 months post-admission (time 2), and 6 months post-admission (time 3) by race/ethnicity.

**Fig. 2.**
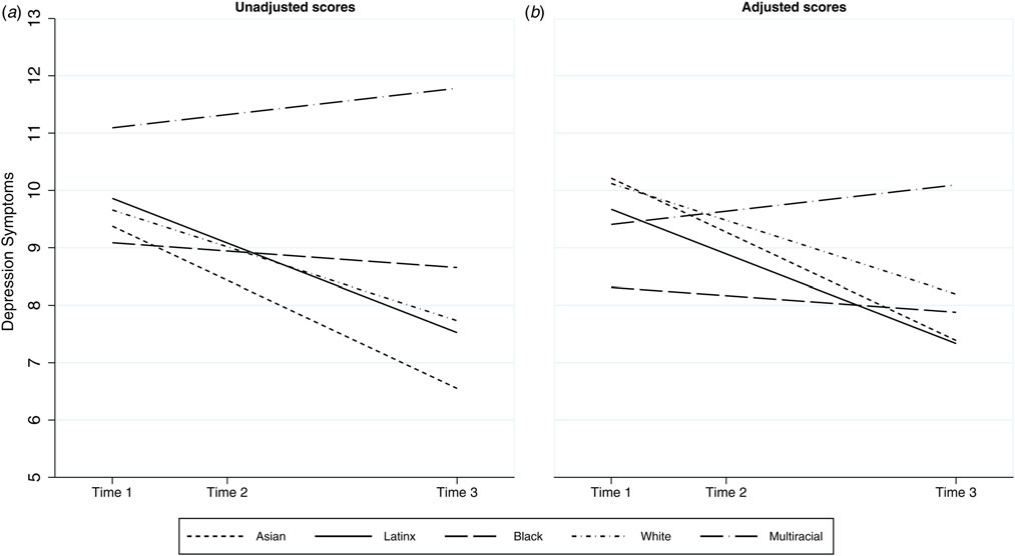
Unadjusted and adjusted mean depression scores at the time of hospitalization (time 1), 2 months post-admission (time 2), and 6 months post-admission (time 3) by race/ethnicity.

**Fig. 3.**
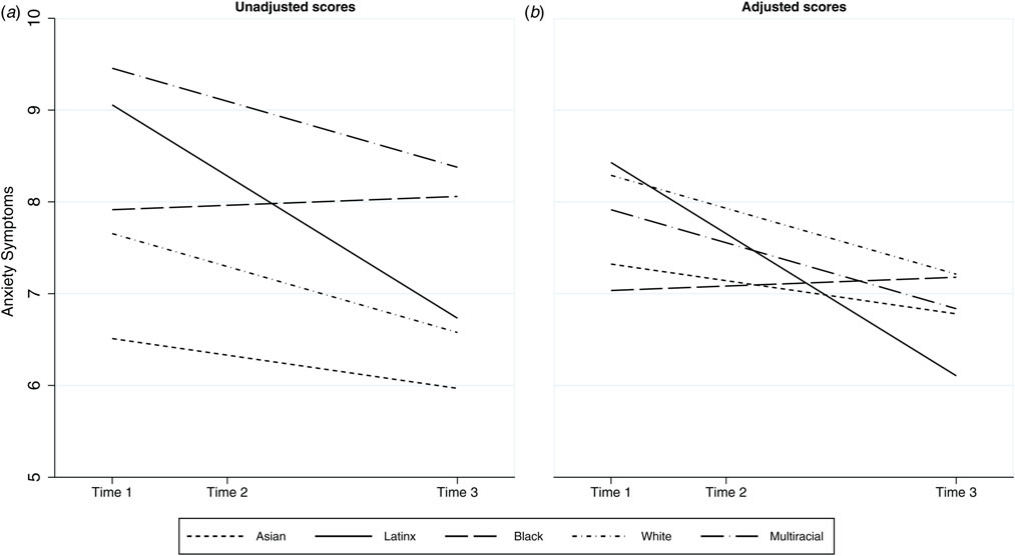
Unadjusted and adjusted mean anxiety scores at the time of hospitalization (time 1), 2 months post-admission (time 2), and 6 months post-admission (time 3) by race/ethnicity.

In unadjusted models, Latinx, Black, and multiracial patients had higher PTSD scores at time 1 than White patients (effect size ‘*d*’ = 0.27, 0.28, and 0.57, respectively) (Table 2 and Fig. 1*a*). Two and 6 months post-admission, PTSD symptoms remained higher among Black (*d* = 0.29–0.34) and multiracial (*d* = 0.49–0.51) but not Latinx patients. No racial/ethnic differences were observed at either time 1, 2, or 3 in depression symptoms (Table 2 and Fig. 2*a*). Latinx and multiracial patients had higher anxiety scores at time 1 than White patients (*d* = 0.21 and 0.27, respectively), which remained higher at time 2 among multiracial (*d* = 0.21) but not Latinx patients. No racial/ethnic differences in anxiety symptoms were observed by time 3 (Table 2 and Fig. 3*a*). After adjusting for pre-, peri-, and post-trauma risk factors, most differences in PTSD symptoms at either time point were no longer observed except for Latinx patients, who had lower PTSD scores at time 3 than White patients (*d* = 0.19) (Table 2 and Fig. 1*b*). In adjusted models, Black patients also had lower depression and anxiety scores at times 1 and 2 than White patients (*d* = 0.10–0.27), but they reached the same levels by time 3 (Table 2 and Figs 2*b* and 3*b*).

**Table 2.** PTSD, depression, and anxiety symptom scores change over time by race/ethnicity in unadjusted models and in models adjusting for risk factors at time 1

Race/ethnicity scores by outcome	Unadjusted scores			Adjusted scores^a^			Average Δ [95% CI]
	Time 1	Time 2	Time 3	Time 1	Time 2	Time 3	
PTSD							
Asian (*N* = 91)	13.94	12.91	10.87	16.15	15.12	13.08	−1.54 [−4.51 to 1.44]
Latinx (*N* = 196)	19.02^‡^	16.45	11.31	17.83	15.26	10.12^‡^	−3.86 [−5.65 to −2.07]*^,‡^
Black (*N* = 310)	19.12^‡^	18.96^‡^	18.63^‡^	16.48	16.32	15.99	−0.24 [−1.52 to 1.03]
Multiracial (*N* = 60)	23.77^‡^	22.99^‡^	21.45^‡^	19.15	18.37	16.83	−1.16 [−4.21 to 1.90]
Referent group: White (*N* = 653)	14.72	13.84	12.08	16.44	15.57	13.81	−1.32 [−2.06 to −0.57]*
Referent group s.d.	(15.86)	(17.79)	(19.29)				(9.74)
Depression							
Asian (*N* = 91)	9.38	8.43	6.55	10.21	9.27	7.39	−1.41 [−2.94 to 0.12]
Latinx (*N* = 196)	9.86	9.08	7.53	9.67	8.89	7.34	−1.17 [−2.14 to −0.19]*
Black (*N* = 310)	9.09	8.94	8.65	8.31^‡^	8.17^‡^	7.88	−0.22 [−0.88 to 0.44]
Multiracial (*N* = 60)	11.09	11.32	11.78	9.41	9.64	10.10	0.34 [−1.25 to 1.94]
Referent group: White (*N* = 653)	9.66	9.02	7.73	10.12	9.48	8.20	−0.96 [−1.34 to −0.59]*
Referent group s.d.	(6.81)	(9.03)	(9.81)				(4.87)
Anxiety							
Asian (*N* = 91)	6.51	6.33	5.97	7.32	7.14	6.78	−0.27 [−1.54 to 0.99]
Latinx (*N* = 196)	9.06^‡^	8.28	6.73	8.43	7.65	6.11	−1.16 [−1.96 to −0.36]*
Black (*N* = 310)	7.91	7.96	8.06	7.03^‡^	7.08^‡^	7.18	0.07 [−0.54 to 0.69]
Multiracial (*N* = 60)	9.46^‡^	9.10^‡^	8.38	7.91	7.55	6.84	−0.54 [−1.94 to 0.86]
Referent group: White (*N* = 653)	7.65	7.30	6.58	8.29	7.93	7.21	−0.54 [−0.89 to −0.18]*
Referent group s.d.	(6.69)	(8.48)	(9.16)				(4.63)

PTSD, posttraumatic stress disorder; CI, confidence interval; s.d., standard deviation of the referent group (in parentheses).

a Models adjusted for pre-, peri-, and post-trauma risk factors at Time 1.

*Statistically significant outcome scores change at the 0.05 level across times.

‡ Statistically significant difference at the 0.05 level compared to White patients, conditional on a significant omnibus test to adjust for multiple comparisons.

On average, PTSD, depression, and anxiety symptoms significantly improved between times 1 and 3 among Latinx and White patients, but were stable among Asian, Black, and multiracial patients (Table 2). Symptom scores change over time was neither greater nor lower among racial/ethnic minority compared to White patients except for PTSD scores, which were on average 3.86 points lower between times 1 and 3 among Latinx patients, and this improvement was greater than the average 1.32-point decrease among White patients (*d* = 0.40). Because risk factors were measured at time 1 only and thus included as time invariant predictors, the rate of symptom scores change over time remained unchanged in adjusted compared to unadjusted models.

### Post-hoc analyses

We examined whether risk factors at time 1 partially explained adjusted differences in PTSD symptoms between Latinx and White patients (online Supplementary Fig. S1) and differences in depression and anxiety symptoms between Black and White patients (online Supplementary Figs S2 and S3). Everyday discrimination, financial stress, past mental health problems, and social constraints were consistently associated with the observed differences. Latinx and White patients with no everyday discrimination, financial stress, past mental health problems, and social constraints had about the same PTSD scores that were stable over time. In contrast, Latinx patients with the highest levels of everyday discrimination, financial stress, past mental health problems, and social constraints had PTSD scores that steadily improved at a faster rate comparted to White patients (online Supplementary Fig. S1). Similarly, Black and White patients with no everyday discrimination, financial stress, past mental health problems, and social constraints had the same depression and anxiety scores. In contrast, Black patients with the highest levels of everyday discrimination, financial stress, past mental health problems, and social constraints had lower depression and anxiety scores at times 1 and 2 than White patients, which reached the same level by time 3 (online Supplementary Figs S2 and S3).

## Discussion

We examined whether differences in pre-, peri-, and post-trauma risk factors explained differences in acute and longer-term PTSD, depression, and anxiety symptoms between racial/ethnic minority and White patients hospitalized after emergency care for severe injury or illness. Specific patterns were consistent with the observed differences in posttraumatic symptoms. Expressly, Latinx, Black, and multiracial patients reported higher levels of risk factors that exacerbate trauma response, including lower socioeconomic status (i.e. lower educational attainment, lower income, and higher levels of financial stress) (Alegría et al., 2013; Brewin, Andrews, & Valentine, 2000), extreme discrimination (Asnaani & Hall-Clark, 2017), and social constraints (Zalta et al., 2021). Latinx patients also reported an elevated peri-traumatic response (subjective trauma severity), which has been associated with an increased risk of PTSD (Alcántara, Casement, & Lewis-Fernández, 2013). These differences might have contributed to elevations in PTSD symptoms at the time of hospitalization among Latinx, Black, and multiracial patients. Furthermore, Black and multiracial patients reported higher levels of additional risk factors that exacerbate trauma response, such as lower expected social support (Zalta et al., 2021) and everyday discrimination (Asnaani & Hall-Clark, 2017; Bird et al., 2021). These additional cumulative risk factors might explain why elevations in PTSD symptoms persisted at 2- and 6-months post-hospitalization in Black and multiracial patients. The attenuation of racial/ethnic differences in PTSD symptoms once risk factors were accounted for suggested that pre-, peri-, and post-trauma risks factors might play causal roles in observed disparities in mental health following severe injury or illness.

Consistent with two prior studies suggesting an absence of racial/ethnic variability in vulnerability to trauma-related psychopathology other than PTSD (McLaughlin et al., 2019) or in the association of stressful life events with later depression and anxiety (Turner & Lloyd, 2004), we did not observe racial/ethnic differences in depression symptoms. Anxiety symptoms were higher among Latinx and multiracial patients at the time of hospitalization compared to White patients, but these differences faded either at 2 months (Latinx patients) or at 6 months post-admission (multiracial patients). After adjusting for risk factors, a significant difference emerged whereby Black patients had lower depression and anxiety symptoms within 2 months post-admission than White patients, a difference no longer observed at 6 months. A prior study examining racial/ethnic differences in posttraumatic stress within 3 months after trauma also found that Black individuals displayed lower depression and anxiety symptoms than White individuals, which was accounted for by differences in prior trauma exposure (Harnett et al., 2022). Our results suggest that differences in depression and anxiety symptoms between Black and White patients in the first 2 months after trauma were not completely accounted for by pre-, peri-, and post-trauma risk factors.

Depression and anxiety symptom trajectory did not differ by race/ethnicity, but the rate of decline in PTSD symptoms was faster in Latinx than White patients. No differences in symptom trajectory were also found in Harnett et al. (2022) study of posttraumatic symptoms within 3 months after trauma, including depression, anxiety, and PTSD symptoms (Harnett et al., 2022). Our results suggest that differences in recovery trajectories beyond 3 months might still not occur for depression and anxiety symptoms (although they may occur beyond the 6-month period captured by the current analyses), but they might occur for PTSD symptoms. Although a faster rate of PTSD symptom improvement did not translate into lower symptoms at either 2 or 6 months among Latinx patients, lower 6-month symptoms emerged after accounting for risk factors. The effect of a faster rate of improvement in PTSD symptoms might thus be magnified after accounting for higher levels of risk factors in Latinx patients. This result is in direct contrast to prior research documenting either no differences in risk or higher risk of developing PTSD symptoms following trauma exposure among Latinx compared to White adults (Alcántara et al., 2013; McLaughlin et al., 2019). A higher risk of PTSD symptoms after trauma might be explained by the fact that prior studies included Latinx adults with high rates of exposure to one of the types of trauma most strongly associated with psychopathology (i.e. physical violence), while we studied Latinx patients experiencing severe injury or illness. Everyday discrimination, financial stress, past mental health problems, and social constraints were four possible mechanisms underlying the lower risk of developing PTSD symptoms 6 months post-trauma. These findings are consistent with prior evidence documenting a significant association between chronic PTSD (i.e. beyond 6 months post-trauma) and racial/ethnic discrimination (Sibrava et al., 2019), social disadvantage (Alcántara et al., 2013), psychiatric history, and post-trauma negative social support (O'Donnell et al., 2008). However, there is limited research regarding racial/ethnic differences in chronic PTSD, with some studies assessing discrimination in only some racial/ethnic groups or not addressing racial/ethnic variation at all. Our results are also consistent with prior literature suggesting that lower vulnerability to trauma-related psychopathology may be the result of greater psychological preparedness among US minority groups who routinely encounter day-to-day and extreme discrimination, stigma, and structural oppression (Kessler, Mickelson, & Williams, 1999; Lewis, Cogburn, & Williams, 2015; Williams, Neighbors, & Jackson, 2008).

Several limitations are worth noting. We studied patients hospitalized after emergency care following severe injury or illness. Our findings may thus not apply to survivors of other types of trauma exposures. Second, posttraumatic symptoms were assessed using self-reported measures. A prior systematic review suggested that variation in conditional risk for PTSD among Latinx relative to White adults was not significant when clinician-administered diagnostic interviews were used in lieu of self-reports (Alcántara et al., 2013). Future research might thus benefit from assessing posttraumatic symptoms using different methods. Third, we had a small sample of Asian and multiracial patients, which limited our power to detect small differences. Prior research has suggested that Asian adults have a lower risk of developing PTSD symptoms after trauma than White adults (Alegría et al., 2013; McLaughlin et al., 2019), a finding not observed in our study. Similarly, although no differences in trauma responses by language were observed, we also had limited power given a small sample of patients who responded to the assessments in Spanish or Mandarin. Due to very small sample size, American Indian or Alaska Native patients were excluded from our analyses, but previous research has demonstrated high rates of trauma exposure and PTSD in American Indian and Alaska Native samples (Bassett, Buchwald, & Manson, 2014). Future work is needed to examine whether the present findings can extend to these minority populations. Data limitations did not allow us to examine racial/ethnic differences by subethnicity and nativity, which have been associated with psychopathology in prior studies (Vilsaint et al., 2019). Finally, there were significant rates of follow-up nonresponse and attrition, which may cause bias and reduce efficiency. However, analyses of missing data patterns made plausible assuming that data were missing at random and the choice of multiple imputation viable. Furthermore, a recent study found that for data missing at random, valid multiple imputation reduces bias even when the proportion of missingness is large (Madley-Dowd, Hughes, Tilling, & Heron, 2019).

Several strengths are also worth noting. We considered a variety of risk factors theoretically related to racial/ethnic disparities in mental health among a diverse cohort of patients with longitudinal data. Study procedures systematically recruited a broad range of patients, including patients with any type of injury or illness, Asian and Multiracial patients, and patients who only spoke Spanish or Mandarin. Prior similar studies have included only traumatic injury patients (Harnett et al., 2022) or motor vehicle crash patients (Ziobrowski et al., 2021). Our findings may thus be applicable to a larger and more diverse population, and provide information about different levels of risk factors potentially related to racial/ethnic differences in posttraumatic symptoms. Lastly, by using imputed data, differences in posttraumatic symptoms were less likely to be associated with race- or ethnicity-related attrition patterns (e.g. Black patients with worse symptoms being more likely to be lost to follow-up than White patients).

The present study showed that although experiencing a potentially traumatic event, such as severe injury or illness, impacted patients' mental health, responses to trauma appeared to differ not by race or ethnicity but by the patients' underlying risk profile. Our results identified pre-trauma exposure to everyday discrimination, financial stress, and past mental health problems and post-trauma exposure to social constraints as salient factors that merit early intervention. Recent work demonstrates that posttraumatic responses in the early aftermath of trauma may be predictive of later trajectories of chronic dysfunction or may map onto other trajectories of gradual recovery and delayed reactions (Bonanno & Mancini, 2012; Galatzer-Levy et al., 2013; Shalev et al., 2019). Thus, our findings not only shed light on pre- and post-trauma risk factors associated with racial/ethnic differences in the acute aftermath of trauma, but also highlight risk factors for which early identification could reduce the debilitating effect of traumatic stressors of severe injury or illness. Screening for mental health risk by brief assessment of these risks could afford an opportunity to inform patients about their risk and be an equitable approach to preventive mental health care.

## Supplementary material

For supplementary material accompanying this paper visit https://doi.org/10.1017/S0033291722002112. click here to view supplementary material

## References

[ref1] Alcántara, C., Casement, M. D., & Lewis-Fernández, R. (2013). Conditional risk for PTSD among Latinos: A systematic review of racial/ethnic differences and sociocultural explanations. Clinical Psychology Review, 33(1), 107–119. 10.1016/j.cpr.2012.10.005. 23159328 PMC3535473

[ref2] Alegría, M., Fortuna, L. R., Lin, J. Y., Norris, F. H., Gao, S., Takeuchi, D. T., … Valentine, A. (2013). Prevalence, risk, and correlates of posttraumatic stress disorder across ethnic and racial minority groups in the United States. Medical Care, 51(12), 1114–1123. 10.1097/MLR.0000000000000007. 24226308 PMC3922129

[ref3] Alegría, M., Pescosolido, B. A., Williams, S., & Canino, G. (2011). Culture, race/ethnicity and disparities: Fleshing out the socio-cultural framework for health services disparities. In B. A. Pescosolido, J. K. Martin, J. D. McLeod & A. Rogers (Eds.), Handbook of the sociology of health, illness, and healing (pp. 363–382). New York, NY: Springer New York. Retrieved from 10.1007/978-1-4419-7261-3_19.

[ref4] Alegria, M., Vila, D., Woo, M., Canino, G., Takeuchi, D., Vera, M., … Shrout, P. (2004). Cultural relevance and equivalence in the NLAAS instrument: Integrating etic and emic in the development of cross-cultural measures for a psychiatric epidemiology and services study of Latinos. International Journal of Methods in Psychiatric Research, 13(4), 270–288. 10.1002/mpr.181. 15719532 PMC2771729

[ref5] Asnaani, A., & Hall-Clark, B. (2017). Recent developments in understanding ethnocultural and race differences in trauma exposure and PTSD. Current Opinion in Psychology, 14, 96–101. 10.1016/j.copsyc.2016.12.005. 28813327

[ref6] Bassett, D., Buchwald, D., & Manson, S. (2014). Posttraumatic stress disorder and symptoms among American Indians and Alaska Natives: A review of the literature. Social Psychiatry and Psychiatric Epidemiology, 49(3), 417–433. 10.1007/s00127-013-0759-y. 24022752 PMC3875613

[ref7] Biering, K., Hjollund, N. H., & Frydenberg, M. (2015). Using multiple imputation to deal with missing data and attrition in longitudinal studies with repeated measures of patient-reported outcomes. Clinical Epidemiology, 91, 91–106. 10.2147/CLEP.S72247. PMC4303367 25653557

[ref8] Bird, C. M., Webb, E. K., Schramm, A. T., Torres, L., Larson, C., & deRoon-Cassini, T. A. (2021). Racial discrimination is associated with acute posttraumatic stress symptoms and predicts future posttraumatic stress disorder symptom severity in trauma-exposed black adults in the United States. Journal of Traumatic Stress, 34(5), 995–1004. 10.1002/jts.22670. 33715212 PMC9123835

[ref9] Bonanno, G. A., & Mancini, A. D. (2012). Beyond resilience and PTSD: Mapping the heterogeneity of responses to potential trauma. Psychological Trauma: Theory, Research, Practice, and Policy, 4(1), 74–83. 10.1037/a0017829.

[ref10] Brewin, C. R., Andrews, B., & Valentine, J. D. (2000). Meta-analysis of risk factors for posttraumatic stress disorder in trauma-exposed adults. Journal of Consulting and Clinical Psychology, 68(5), 748–766. 10.1037/0022-006X.68.5.748. 11068961

[ref11] Carlson, E. B. (2001). Psychometric study of a brief screen for PTSD: Assessing the impact of multiple traumatic events. Assessment, 8(4), 431–441. 10.1177/107319110100800408. 11785587

[ref12] Carlson, E. B., Dalenberg, C. J., & Muhtadie, L. (2008). The etiology of posttraumatic stress disorder. In G. Reyes, J. D. Elhai, & J. D. Ford (Eds.), The encyclopedia of psychological trauma (pp. 257–264). Hoboken, N.J.: Wiley.

[ref13] Carlson, E. B., Palmieri, P. A., Field, N. P., Dalenberg, C. J., Macia, K. S., & Spain, D. A. (2016). Contributions of risk and protective factors to prediction of psychological symptoms after traumatic experiences. Comprehensive Psychiatry, 69, 106–115. 10.1016/j.comppsych.2016.04.022. 27423351 PMC5381967

[ref14] Carlson, E. B., Palmieri, P. A., & Spain, D. A. (2017). Development and preliminary performance of a risk factor screen to predict posttraumatic psychological disorder after trauma exposure. General Hospital Psychiatry, 46, 25–31. 10.1016/j.genhosppsych.2016.12.011. 28622811 PMC5656435

[ref15] Davis, R. G., Ressler, K. J., Schwartz, A. C., Stephens, K. J., & Bradley, R. G. (2008). Treatment barriers for low-income, urban African Americans with undiagnosed posttraumatic stress disorder. Journal of Traumatic Stress, 21(2), 218–222. 10.1002/jts.20313. 18404649 PMC2444044

[ref16] Eitle, D., & Turner, R. J. (2002). Exposure to community violence and young adult crime: The effects of witnessing violence, traumatic victimization, and other stressful life events. Journal of Research in Crime and Delinquency, 39(2), 214–237. 10.1177/002242780203900204.

[ref17] Gaffey, A. E., Aranda, F., Burns, J. W., Purim-Shem-Tov, Y. A., Burgess, H. J., Beckham, J. C., … Hobfoll, S. E. (2019). Race, psychosocial vulnerability and social support differences in inner-city women's symptoms of posttraumatic stress disorder. Anxiety, Stress, & Coping, 32(1), 18–31. 10.1080/10615806.2018.1532078. 30306795 PMC6269211

[ref18] Galatzer-Levy, I. R., Ankri, Y., Freedman, S., Israeli-Shalev, Y., Roitman, P., Gilad, M., & Shalev, A. Y. (2013). Early PTSD symptom trajectories: Persistence, recovery, and response to treatment: Results from the Jerusalem trauma outreach and prevention study (J-TOPS). PLoS ONE, 8(8), e70084. 10.1371/journal.pone.0070084. 23990895 PMC3750016

[ref19] Grant, D. M., Beck, J. G., Marques, L., Palyo, S. A., & Clapp, J. D. (2008). The structure of distress following trauma: Posttraumatic stress disorder, major depressive disorder, and generalized anxiety disorder. Journal of Abnormal Psychology, 117(3), 662–672. 10.1037/a0012591. 18729617

[ref20] Harnett, N. G., Dumornay, N. M., Delity, M., Sanchez, L. D., Mohiuddin, K., Musey, P. I., … Ressler, K. J. (2022). Prior differences in previous trauma exposure primarily drive the observed racial/ethnic differences in posttrauma depression and anxiety following a recent trauma. Psychological Medicine, 1–10. 10.1017/S0033291721004475. PMC9339026 35094717

[ref21] Holzer, C. E., & Copeland, S. (2013). Race, ethnicity, and the epidemiology of mental disorders in adults. In F. A. Paniagua, & A.-M. Yamada (Eds.), Handbook of multicultural mental health (pp. 89–109). San Diego, CA: Elsevier. 10.1016/B978-0-12-394420-7.00005-9.

[ref22] Jackson, J. S., Knight, K. M., & Rafferty, J. A. (2010). Race and unhealthy behaviors: Chronic stress, the HPA axis, and physical and mental health disparities over the life course. American Journal of Public Health, 100(5), 933–939. 10.2105/AJPH.2008.143446. 19846689 PMC2853611

[ref23] Kessler, R. C., Mickelson, K. D., & Williams, D. R. (1999). The prevalence, distribution, and mental health correlates of perceived discrimination in the United States. Journal of Health and Social Behavior, 40(3), 208. 10.2307/2676349. 10513145

[ref24] Kim, G., Sellbom, M., & Ford, K.-L. (2014). Race/ethnicity and measurement equivalence of the Everyday Discrimination Scale. Psychological Assessment, 26(3), 892–900. 10.1037/a0036431. 24708076 PMC4152383

[ref25] Kroenke, K., & Spitzer, R. L. (2002). The PHQ-9: A new depression diagnostic and severity measure. Psychiatric Annals, 32(9), 509–515. 10.3928/0048-5713-20020901-06.

[ref26] Lepore, S., & Ituarte, P. (1999). Optimism about cancer enhances mood by reducing negative social relations. Cancer Research, Therapy and Control, 8(3), 165–174.

[ref27] Lewis, T. T., Cogburn, C. D., & Williams, D. R. (2015). Self-reported experiences of discrimination and health: Scientific advances, ongoing controversies, and emerging issues. Annual Review of Clinical Psychology, 11(1), 407–440. 10.1146/annurev-clinpsy-032814-112728. PMC5555118 25581238

[ref28] Madley-Dowd, P., Hughes, R., Tilling, K., & Heron, J. (2019). The proportion of missing data should not be used to guide decisions on multiple imputation. Journal of Clinical Epidemiology, 110, 63–73. 10.1016/j.jclinepi.2019.02.016. 30878639 PMC6547017

[ref29] McLaughlin, K. A., Alvarez, K., Fillbrunn, M., Green, J. G., Jackson, J. S., Kessler, R. C., … Alegría, M. (2019). Racial/ethnic variation in trauma-related psychopathology in the United States: A population-based study. Psychological Medicine, 49(13), 2215–2226. 10.1017/S0033291718003082. 30378513 PMC6494744

[ref30] O'Donnell, M. L., Creamer, M. C., Parslow, R., Elliott, P., Holmes, A. C. N., Ellen, S., … Bryant, R. A. (2008). A predictive screening index for posttraumatic stress disorder and depression following traumatic injury. Journal of Consulting and Clinical Psychology, 76(6), 923–932. 10.1037/a0012918. 19045961

[ref31] RStudio Team. (2021). RStudio: Integrated development environment for R *(*version 1.4.1717*)*. Boston, MA: RStudio, PBC. Retrieved from http://www.rstudio.com/.

[ref32] Rui, P., Kang, K., & Albert, M. (2013). *National hospital ambulatory medical care survey: Emergency department summary tables*. Retrieved from http://www.cdc.gov/nchs/data/ahcd/nhamcs_emergency/2013_ed_web_tables.pdf.

[ref33] Santos, M. R., Russo, J., Aisenberg, G., Uehara, E., Ghesquiere, A., & Zatzick, D. F. (2008). Ethnic/racial diversity and posttraumatic distress in the acute care medical setting. Psychiatry: Interpersonal and Biological Processes, 71(3), 234–245. 10.1521/psyc.2008.71.3.234. 18834274

[ref34] Shalev, A. Y., Gevonden, M., Ratanatharathorn, A., Laska, E., van der Mei, W. F., Qi, W., … van Zuiden, M. (2019). Estimating the risk of PTSD in recent trauma survivors: Results of the International Consortium to Predict PTSD (ICPP). World Psychiatry, 18(1), 77–87. 10.1002/wps.20608. 30600620 PMC6313248

[ref35] Sherbourne, C. D., & Stewart, A. L. (1991). The MOS social support survey. Social Science & Medicine, 32(6), 705–714. 10.1016/0277-9536(91)90150-B. 2035047

[ref36] Sibrava, N. J., Bjornsson, A. S., Pérez Benítez, A. C. I., Moitra, E., Weisberg, R. B., & Keller, M. B. (2019). Posttraumatic stress disorder in African American and Latinx adults: Clinical course and the role of racial and ethnic discrimination. American Psychologist, 74(1), 101–116. 10.1037/amp0000339. 30652903 PMC6338337

[ref37] Spitzer, R. L., Kroenke, K., Williams, J. B. W., & Löwe, B. (2006). A brief measure for assessing generalized anxiety disorder: The GAD-7. Archives of Internal Medicine, 166(10), 1092. 10.1001/archinte.166.10.1092. 16717171

[ref38] StataCorp. (2017). Stata statistical software: Release 15 *(*version 15*)*. College Station, TX: StataCorp LLC.

[ref39] Stephens, K. A., Sue, S., Roy-Byrne, P., Unützer, J., Wang, J., Rivara, F. P., … Zatzick, D. F. (2010). Ethnoracial variations in acute PTSD symptoms among hospitalized survivors of traumatic injury. Journal of Traumatic Stress, 23(3), 384–392. 10.1002/jts.20534. 20564368 PMC3947745

[ref40] Torres, L., & Taknint, J. T. (2015). Ethnic microaggressions, traumatic stress symptoms, and Latino depression: A moderated mediational model. Journal of Counseling Psychology, 62(3), 393–401. 10.1037/cou0000077. 25867692

[ref41] Turner, R. J., & Lloyd, D. A. (2004). Stress burden and the lifetime incidence of psychiatric disorder in young adults: Racial and ethnic contrasts. Archives of General Psychiatry, 61(5), 481. 10.1001/archpsyc.61.5.481. 15123493

[ref42] Utsey, S. O., & Ponterotto, J. G. (1996). Development and validation of the Index of Race-Related Stress (IRRS). Journal of Counseling Psychology, 43(4), 490–501. 10.1037/0022-0167.43.4.490.

[ref43] Vilsaint, C. L., NeMoyer, A., Fillbrunn, M., Sadikova, E., Kessler, R. C., Sampson, N. A., … Alegría, M. (2019). Racial/ethnic differences in 12-month prevalence and persistence of mood, anxiety, and substance use disorders: Variation by nativity and socioeconomic status. Comprehensive Psychiatry, 89, 52–60. 10.1016/j.comppsych.2018.12.008. 30594752 PMC6421861

[ref44] Waelde, L. C., Pennington, D., Mahan, C., Mahan, R., Kabour, M., & Marquett, R. (2010). Psychometric properties of the Race-Related Events Scale. Psychological Trauma: Theory, Research, Practice, and Policy, 2(1), 4–11. 10.1037/a0019018.

[ref45] Williams, D. R., & Mohammed, S. A. (2009). Discrimination and racial disparities in health: Evidence and needed research. Journal of Behavioral Medicine, 32(1), 20–47. 10.1007/s10865-008-9185-0. 19030981 PMC2821669

[ref46] Williams, D. R., Neighbors, H. W., & Jackson, J. S. (2008). Racial/ethnic discrimination and health: Findings from community studies. American Journal of Public Health, 98(Supplement_1), S29–S37. 10.2105/AJPH.98.Supplement_1.S29. 18687616 PMC2518588

[ref47] Zalta, A. K., Tirone, V., Orlowska, D., Blais, R. K., Lofgreen, A., Klassen, B., … Dent, A. L. (2021). Examining moderators of the relationship between social support and self-reported PTSD symptoms: A meta-analysis. Psychological Bulletin, 147(1), 33–54. 10.1037/bul0000316. 33271023 PMC8101258

[ref48] Zatzick, D. F., Rivara, F. P., Nathens, A. B., Jurkovich, G. J., Wang, J., Fan, M.-Y., … Mackenzie, E. J. (2007). A nationwide US study of post-traumatic stress after hospitalization for physical injury. Psychological Medicine, 37(10), 1469–1480. 10.1017/S0033291707000943. 17559704

[ref49] Ziobrowski, H. N., Kennedy, C. J., Ustun, B., House, S. L., Beaudoin, F. L., An, X., … van Rooij, S. J. H. (2021). Development and validation of a model to predict posttraumatic stress disorder and major depression after a motor vehicle collision. JAMA Psychiatry, 78(11), 1228. 10.1001/jamapsychiatry.2021.2427. 34468741 PMC8411364

