# Supplementary material for: Racial/ethnic differences in acute and longer-term posttraumatic symptoms following traumatic injury or illness
Source: Psychol Med. 2022 Jul 29;53(11):5099–108. doi: 10.1017/S0033291722002112 (PMC9884321; doi:10.1017/S0033291722002112)
Supplement: Supplementary file 1 [file S0033291722002112sup001.docx]

**Racial/ethnic differences in acute and longer-term posttraumatic stress symptoms following traumatic injury or illness: Online supplementary material**

**1. Supplementary information on missing data**

From 1,310 patients included in the present study that completed the Time 1 assessment, 491 (37.48%) completed all follow-up assessments, 298 (22.75%) completed the Time 2 follow-up assessment only, 87 (6.64%) completed the Time 3 follow-up assessment only, and 434 (33.13%) were lost to follow-up at both Time 2 and Time 3. Although we observed racial/ethnic differences in response rates post-hospitalization [χ^2^(12) = 109.68, *p* < .01], our study focused on the association between race/ethnicity and changes in traumatic stress response over time. Thus, we examined the potential impact of missing outcome follow-up data by comparing both, responses to traumatic stress and risk factors at Time 1, between patients who completed all follow-up assessments and patients who missed at least one follow-up assessment within each racial/ethnic group. As shown in Table S2 of the present Supplement, no differences in risk factors were observed in Latinx patients, and very few risk factors were significantly different within the other racial/ethnic groups. We also did not observe significant differences in posttraumatic symptoms at Time 1 among Asian and Latinx patients. Within Black patients, those who completed all three assessments reported lower PTSD and depression symptoms but the same anxiety symptoms. Among White patients, significant differences were observed for PTSD and anxiety symptoms, while for multiracial patients, significant differences were observed for PTSD symptoms. After adjusting for pre-trauma, time-of-trauma, and post-trauma risk factors, no differences in posttraumatic symptoms were longer observed among Black and multiracial patients, and only PTSD symptoms remained significantly lower in patients that completed all three assessments among Whites. This result suggested that we could reasonably assume that risk factors at Time 1 were associated with follow-up nonresponse and attrition, and thus could be used as explanatory variables in a stochastic model to predict missing values using multiple imputation.

We followed Biering, Hjollund, and Frydenberg’s (2015) application of multiple imputation in a follow-up study of repeated patient-reported measures with high rates of missing data (Biering, Hjollund, & Frydenberg, 2015). We began by specifying a stochastic model for each variable with missing values. Regarding the outcomes, we used results from our previous study (Carlson et al., 2016) showing that 72% of the variance in posttraumatic symptoms (including symptoms of PTSD and depression) was accounted for by a linear regression model that included most of the pre-, time-of-, and post-trauma risk factors used in the present study. Thus, for each outcome $g$ at time $t$, $y_{g,t}$, we imputed values according to the following linear regression model:

$$y_{g,t}=\alpha_{1}y_{g,t+1}+\alpha_{2}y_{g,t-1}+\boldsymbol{\alpha}_{3}y_{\sim g,t}+\boldsymbol{\alpha}_{4}y_{\sim g,t-1}+\boldsymbol{\alpha}_{5}\left( race/ethnicity \right)$$

$$\begin{aligned} +\boldsymbol{\alpha}_{6}\left( risk factors \right)+\alpha_{7}(illness/injury)+hospital_{\gamma}\#\left( 1 \right) \end{aligned}$$

where $t+1$ and $t-1$ represent the following and previous assessment, respectively; $\sim g$ is a vector that includes all other outcomes but outcome $g$; $race/ethnicity$ is a categorical variable that indicates whether the observed value belongs to a patient who self-identified as either Asian, Latinx, Black, White, or multiracial; $risk factors$ is the vector of pre-, time-of-, and post-trauma risk factors at Time 1; $illness/injury$ is an indicator variable of whether a patient was hospitalized because of traumatic injury or severe illness, and $hospital_{\gamma}$ are hospital fixed-effects. We noted that the value $t+1$ does not exist for Time 3, while the value $t-1$ does not exist for Time 1. The linear model from Equation $(1)$ indicates that outcome scores $g$ at time $t$ depend on [1] its previous ($y_{g,t-1}$) and following scores ($y_{g,t+1}$); [2] the previous ($y_{\sim g,t-1}$) and current scores ($y_{\sim g,t}$) of the other outcomes; [3] a patient’s race/ethnicity; and [4] risk factors at Time 1. Using PTSD symptoms at Time 2 as an example, the linear regression model from Equation $(1)$ would be as follows:

$$PTSD_{2}=\alpha_{1}PTSD_{3}+\alpha_{2}PTSD_{1}+\boldsymbol{\alpha}_{3}\left( Depression_{2},Anxiety_{2} \right)+\boldsymbol{\alpha}_{4}(Depression_{1},Anxiety_{1})+\boldsymbol{\alpha}_{5}\left( race/ethnicity \right)+\boldsymbol{\alpha}_{6}\left( risk factors \right)+\alpha_{7}(illness/injury)+hospital_{\gamma}$$

Risks factors at Time 1 that did not vary over time were modeled using a very similar linear model but including only variables measured at Time 1. Using everyday discrimination as an example, the linear model used to impute missing values for risk factors at Time 1 would look as follows:

$$Everyday discrimination=\boldsymbol{\beta}_{1}\left( PTSD_{1},Depression_{1},Anxiety_{1} \right)+\boldsymbol{\beta}_{2}\left( race/ethnicity \right)+\boldsymbol{\beta}_{3}\left( risk factors other than everyday discrimination \right)+\beta_{4}(illness/injury)+hospital_{\gamma}$$

We created 50 imputed datasets using multiple imputation by chained equations in the Stata software version 15 (StataCorp, 2017).

**2. Supplementary information on individual growth curve models**

Since we were interested in describing the change over time in posttraumatic stress symptoms during our 6-month follow-up period, we estimated individual growth curve models using follow-up time at the participant-level as the relevant “time” variable. In our study, we could easily conceptualize our growth curve model as a two-level model. The Level 1 or within-participant model captures individual-specific growth rates; the “time” variable is thus included at this level. The Level 2 or between-participant model captures between-individual growth rate variability; time-invariant predictors (in our case race/ethnicity and all risk factors at Time 1) are included at this level. We hypothesized that race/ethnicity would be associated with both differing symptoms at the time of hospitalization (i.e., differences in intercepts) and differing growth rates of symptoms (i.e., differences in slopes) at 2- and 6-months post-admission, and that these differences would be accounted for by risk factors at Time 1. The equations representing this conceptualization are given by:

$$\begin{aligned} \text{Level 1}\text{:} y_{ij}=b_{0i}+b_{1i}\left( time_{ij} \right)+e_{ij} \#\left( 1a \right) \end{aligned}$$

$$\begin{aligned} \text{Level 2}\text{:} b_{0i}=b_{00}+\boldsymbol{b}_{01}^{'}\left( race/ethnicity_{i}^{k} \right)+\boldsymbol{b}_{02}^{'}\left( risk factors_{i} \right)+u_{0i}\#\left( 1b \right) \end{aligned}$$

$$\begin{aligned} b_{1i}=b_{10}+\boldsymbol{b}_{11}^{'}\left( race/ethnicity_{i}^{k} \right)+u_{1i} \#\left( 1c \right) \end{aligned}$$

where $y_{ij}$ is an outcome variable from participant $i$ at measurement occasion $j$; $time_{ij}$ represents follow-up time for participant $i$ at measurement occasion $j$ (coded 0 for Time 1, and 2 and 6 for Times 2 and 3, respectively); $race/ethnicity_{i}^{k}$ measures whether participant $i$ self-identified in racial/ethnic minority group $k$ (Asian, Latinx, Black, White, and multiracial); $risk factors_{i}$ is the vector of pre-, time-of-, and post-trauma risk factors at Time 1 from participant $i$; and $e_{ij}$ is a residual.

At Level 1, $b_{0i}$ and $b_{1i}$ represent the within-participant intercept and slope, respectively (i.e., both parameters are allowed to vary across participants). At Level 2, each participant’s intercept, $b_{0i}$, is a function of four parameters: the population intercept ($b_{00})$, their race/ethnicity ($\boldsymbol{b}_{01})$, their levels of risk factors at Time 1 ($\boldsymbol{b}_{02})$, and their individual deviations from the population intercept ($u_{0i})$. Similarly, each participant’s slope, $b_{1i}$, is a function of three parameters: the population slope ($b_{10})$, their race/ethnicity ($\boldsymbol{b}_{11})$, and their individual deviations from the population slope ($u_{1i})$. Substituting the Level 2 model into the Level 1 model, our final model was reduced to:

$$y_{ij}=b_{00}+\boldsymbol{b}_{01}^{'}\left( race/ethnicity_{i}^{k} \right)+\boldsymbol{b}_{02}^{'}\left( risk factors_{i} \right)+b_{10}\left( time_{ij} \right)+$$

$$\begin{aligned} +\boldsymbol{b}_{11}^{'}\left( race/ethnicity_{i}^{k}\times time_{ij} \right)+u_{0i}+u_{1i}\left( time_{ij} \right)+e_{ij} \#\left( 2 \right) \end{aligned}$$

The parameter $\boldsymbol{b}_{01}$ measures whether participants of different race/ethnicity exhibited differing symptoms at the time of hospitalization (i.e., differences in intercepts), while the parameter $\boldsymbol{b}_{11}$ measures whether there were racial/ethnic differences in outcome trajectory (i.e., differences in slopes).

In post-hoc analyses, we expanded our two-level model to examine whether levels of risk factors at Time 1 partially explained racial/ethnic differences in intercepts and slopes; that is, the Level 2 model was expanded to include two-way interactions between race/ethnicity and risk factors as predictors of both intercept and slope variability:

$$\begin{aligned} \text{Level 1}\text{:} y_{ij}=b_{0i}+b_{1i}\left( time_{ij} \right)+e_{ij} \#\left( 3a \right) \end{aligned}$$

$$\text{Level 2}\text{:} b_{0i}=b_{00}+\boldsymbol{b}_{01}^{'}\left( race/ethnicity_{i}^{k} \right)+\boldsymbol{b}_{02}^{'}\left( risk factors_{i} \right)+$$

$$\begin{aligned} +\boldsymbol{b}_{03}^{'}\left( race/ethnicity_{i}^{k}\times risk factors_{i} \right)+u_{0i} \#\left( 3b \right) \end{aligned}$$

$$b_{1i}=b_{10}+\boldsymbol{b}_{11}^{'}\left( race/ethnicity_{i}^{k} \right)+\boldsymbol{b}_{12}^{'}\left( risk factors_{i} \right)+$$

$$\begin{aligned} +\boldsymbol{b}_{13}^{'}\left( race/ethnicity_{i}^{k}\times risk factors_{i} \right)+u_{1i} \#\left( 3c \right) \end{aligned}$$

To avoid model overspecification, we included two-way interactions between race/ethnicity and risk factors at Time 1 one at a time. The parameter $\boldsymbol{b}_{03}$ measures whether participants of different race/ethnicity exhibited differing symptoms at the time of hospitalization as a function of their Time 1 levels of risk factors, while the parameter $\boldsymbol{b}_{13}$ measures whether racial/ethnic differences in outcome trajectory were a function of risk factors at Time 1.

**References**

Biering, K., Hjollund, N. H., & Frydenberg, M. (2015). Using multiple imputation to deal with missing data and attrition in longitudinal studies with repeated measures of patient-reported outcomes. *Clinical Epidemiology*, 91. https://doi.org/10.2147/CLEP.S72247

Carlson, E. B., Palmieri, P. A., Field, N. P., Dalenberg, C. J., Macia, K. S., & Spain, D. A. (2016). Contributions of risk and protective factors to prediction of psychological symptoms after traumatic experiences. *Comprehensive Psychiatry*, *69*, 106–115. https://doi.org/10.1016/j.comppsych.2016.04.022

StataCorp. (2017). Stata Statistical Software: Release 15 (Version 15). College Station, TX: StataCorp LLC.

| **Table S1. Internal consistency (Cronbach's α) of study measures by race/ethnicity.** | | | | | | |  |
| --- | --- | --- | --- | --- | --- | --- | --- |
|  | Overall | Asian | Latinx | Black | Multiracial | White | |
| PTSD symptoms |  |  |  |  |  |  | |
| Time 1 | 0.93 | 0.92 | 0.93 | 0.93 | 0.95 | 0.92 | |
| Time 2 | 0.93 | 0.92 | 0.92 | 0.94 | 0.91 | 0.93 | |
| Time 3 | 0.94 | 0.91 | 0.93 | 0.94 | 0.97 | 0.94 | |
| Depression symptoms |  |  |  |  |  |  | |
| Time 1 | 0.81 | 0.82 | 0.81 | 0.82 | 0.80 | 0.80 | |
| Time 2 | 0.89 | 0.89 | 0.83 | 0.89 | 0.84 | 0.90 | |
| Time 3 | 0.92 | 0.85 | 0.93 | 0.92 | 0.95 | 0.92 | |
| Anxiety symptoms |  |  |  |  |  |  | |
| Time 1 | 0.90 | 0.89 | 0.91 | 0.89 | 0.89 | 0.90 | |
| Time 2 | 0.93 | 0.90 | 0.93 | 0.92 | 0.91 | 0.94 | |
| Time 3 | 0.95 | 0.93 | 0.91 | 0.94 | 0.98 | 0.96 | |
| Everyday discrimination | 0.90 | 0.91 | 0.92 | 0.90 | 0.91 | 0.90 | |
| Expected social support | 0.96 | 0.96 | 0.98 | 0.96 | 0.96 | 0.96 | |
| Social constraints | 0.86 | 0.84 | 0.82 | 0.88 | 0.83 | 0.87 | |

| **Table S2. Attrition patterns for responses to traumatic stress and risk variables at the time of hospitalization (Time 1) by race/ethnicity.** | | | | | | | | | | | |
| --- | --- | --- | --- | --- | --- | --- | --- | --- | --- | --- | --- |
| **Variable at Time 1** | **Asian (n = 91)**  **all waves** | | | | **Latinx (n = 196)**  **all waves** | | | | **Black (n = 310)**  **all waves** | | |
|  | No  (n = 75) | Yes  (n = 16) | *P* | No  (n = 148) | | Yes  (n = 48) | *P* | No  (n = 214) | | Yes  (n = 96) | *P* |
|  | mean (SD) | mean (SD) |  | mean (SD) | | mean (SD) |  | mean (SD) | | mean (SD) |  |
| Outcome measures |  |  |  |  | |  |  |  | |  |  |
| PTSD symptoms | 13.1 (15.1) | 16.6 (10.1) | .39 | 20.6 (17.1) | | 20.3 (15.4) | .89 | 22.6 (18.5) | | 17.0 (18.0) | .01 |
| Depression | 9.6 (7.5) | 11.5 (7.3) | .35 | 10.3 (6.2) | | 10.6 (5.8) | .82 | 10.2 (7.1) | | 8.5 (6.4) | .05 |
| Anxiety | 5.9 (5.9) | 8.6 (6.8) | .10 | 9.2 (6.1) | | 9.9 (6.6) | .51 | 9.1 (6.5) | | 8.0 (6.5) | .18 |
| Pre-trauma factors |  |  |  |  | |  |  |  | |  |  |
| Demographics |  |  |  |  | |  |  |  | |  |  |
| Gender |  |  |  |  | |  |  |  | |  |  |
| Female | 0.5 (0.5) | 0.6 (0.5) | .34 | 0.4 (0.5) | | 0.4 (0.5) | .71 | 0.4 (0.5) | | 0.6 (0.5) | <.01 |
| Male | 0.5 (0.5) | 0.4 (0.5) |  | 0.6 (0.5) | | 0.6 (0.5) |  | 0.6 (0.5) | | 0.4 (0.5) |  |
| Age | 49.0 (18.8) | 42.1 (17.4) | .19 | 44.0 (17.0) | | 46.5 (14.0) | .34 | 43.6 (15.3) | | 47.1 (13.6) | .053 |
| Marital status |  |  |  |  | |  |  |  | |  |  |
| Never married | 0.3 (0.4) | 0.3 (0.4) | .29 | 0.3 (0.5) | | 0.2 (0.4) | .33 | 0.4 (0.5) | | 0.4 (0.5) | .61 |
| Married/cohabitating | 0.6 (0.5) | 0.6 (0.5) |  | 0.4 (0.5) | | 0.5 (0.5) |  | 0.3 (0.5) | | 0.3 (0.5) |  |
| Separated/divorced/widowed | 0.1 (0.3) | 0.1 (0.3) |  | 0.2 (0.4) | | 0.3 (0.4) |  | 0.2 (0.4) | | 0.2 (0.4) |  |
| Other | 0.0 (0.0) | 0.1 (0.3) |  | 0.0 (0.1) | | 0.0 (0.0) |  | 0.0 (0.2) | | 0.0 (0.2) |  |
| Missing | 0.0 (0.1) | 0.0 (0.0) |  | 0.0 (0.2) | | 0.0 (0.0) |  | 0.0 (0.2) | | 0.0 (0.1) |  |
| Education level |  |  |  |  | |  |  |  | |  |  |
| 0-11 years | 0.1 (0.3) | 0.1 (0.3) | .37 | 0.4 (0.5) | | 0.4 (0.5) | .85 | 0.1 (0.4) | | 0.1 (0.3) | .32 |
| 12 years (HS) | 0.1 (0.4) | 0.0 (0.0) |  | 0.2 (0.4) | | 0.2 (0.4) |  | 0.3 (0.5) | | 0.3 (0.5) |  |
| 13-15 years | 0.2 (0.4) | 0.3 (0.4) |  | 0.3 (0.5) | | 0.3 (0.5) |  | 0.4 (0.5) | | 0.4 (0.5) |  |
| 16+ years | 0.6 (0.5) | 0.6 (0.5) |  | 0.1 (0.2) | | 0.1 (0.3) |  | 0.1 (0.3) | | 0.2 (0.4) |  |
| (*continued*) | | | | | | | | | | | |

| **Table S2. Attrition patterns for responses to traumatic stress and risk variables at the time of hospitalization (Time 1) by race/ethnicity.** | | | | | | | | | |
| --- | --- | --- | --- | --- | --- | --- | --- | --- | --- |
| **Variable at Time 1** | **Asian (n = 91)**  **all waves** | | | **Latinx (n = 196)**  **all waves** | | | **Black (n = 310)**  **all waves** | | |
|  | No  (n = 75) | Yes  (n = 16) | *P* | No  (n = 148) | Yes  (n = 48) | *P* | No  (n = 214) | Yes  (n = 96) | *P* |
|  | mean (SD) | mean (SD) |  | mean (SD) | mean (SD) |  | mean (SD) | mean (SD) |  |
| Household income |  |  |  |  |  |  |  |  |  |
| Less than $35k | 0.2 (0.4) | 0.1 (0.3) | .57 | 0.2 (0.4) | 0.3 (0.5) | .75 | 0.5 (0.5) | 0.6 (0.5) | .39 |
| $35K to less than $75k | 0.1 (0.2) | 0.1 (0.3) |  | 0.1 (0.3) | 0.2 (0.4) |  | 0.2 (0.4) | 0.2 (0.4) |  |
| $75K and more | 0.4 (0.5) | 0.6 (0.5) |  | 0.1 (0.3) | 0.1 (0.2) |  | 0.1 (0.2) | 0.1 (0.2) |  |
| Don't know/refused | 0.3 (0.5) | 0.2 (0.4) |  | 0.5 (0.5) | 0.5 (0.5) |  | 0.3 (0.4) | 0.2 (0.4) |  |
| Early life |  |  |  |  |  |  |  |  |  |
| Childhood home life | 3.6 (1.3) | 3.4 (1.2) | .54 | 3.4 (1.2) | 3.2 (1.2) | .32 | 3.4 (1.2) | 3.4 (1.2) | .87 |
| Caretaker dysfunction | 0.3 (0.7) | 0.2 (0.4) | .59 | 0.8 (1.2) | 0.7 (0.9) | .64 | 0.7 (1.1) | 0.6 (1.0) | .40 |
| Past trauma exposure |  |  |  |  |  |  |  |  |  |
| Lifetime sudden/terrible events ^a^ | 3.7 (7.0) | 10.6 (16.5) | <.01 | 4.2 (6.6) | 5.0 (9.5) | .52 | 5.2 (7.8) | 5.0 (7.8) | .86 |
| Perceived discrimination |  |  |  |  |  |  |  |  |  |
| Everyday discrimination | 6.2 (8.1) | 7.0 (6.1) | .71 | 5.5 (8.1) | 5.0 (7.2) | .75 | 9.9 (10.2) | 9.2 (8.5) | .52 |
| Extreme discrimination | 1.0 (2.1) | 2.4 (2.9) | .02 | 2.2 (3.3) | 1.7 (2.8) | .36 | 2.7 (3.4) | 2.1 (2.6) | .16 |
| Financial stress | 2.3 (1.9) | 2.0 (1.8) | .51 | 4.1 (2.3) | 3.8 (1.9) | .54 | 4.6 (2.2) | 4.2 (2.1) | .11 |
| Past mental health problems ^b^ | 1.1 (1.0) | 1.5 (1.3) | .16 | 1.1 (1.1) | 1.1 (0.9) | .85 | 1.1 (1.1) | 1.2 (1.3) | .73 |
| Peri-trauma factors |  |  |  |  |  |  |  |  |  |
| Subjective trauma severity ^c^ | 3.8 (2.1) | 4.2 (2.0) | .53 | 4.4 (1.9) | 4.2 (1.8) | .49 | 4.0 (2.0) | 3.4 (2.3) | .04 |
| Post-trauma factors |  |  |  |  |  |  |  |  |  |
| Expected social support | 24.1 (8.5) | 24.9 (8.2) | .76 | 23.2 (9.0) | 21.6 (10.2) | .29 | 21.5 (9.4) | 22.3 (9.2) | .48 |
| Social constraints | 4.3 (6.3) | 2.6 (3.7) | .31 | 6.0 (6.6) | 6.5 (6.1) | .62 | 5.6 (7.3) | 4.4 (6.5) | .15 |
| (*continued*) | | | | | | | | | |

| **Table S2. Attrition patterns for responses to traumatic stress and risk variables at the time of hospitalization (Time 1) by race/ethnicity.** | | | | | | |
| --- | --- | --- | --- | --- | --- | --- |
| **Variable at Time 1** | **White (n = 654) all waves** | | | **Multiracial (n = 60) all waves** | | |
|  | No (n = 341) | Yes (n = 312) | *P* | No (n = 41) | Yes (n = 19) | *P* |
|  | mean (SD) | mean (SD) |  | mean (SD) | mean (SD) |  |
| Outcome measures |  |  |  |  |  |  |
| PTSD symptoms | 18.1 (17.1) | 12.6 (13.9) | <.01 | 29.6 (25.9) | 15.4 (13.3) | .03 |
| Depression | 10.4 (6.7) | 9.9 (6.9) | .39 | 11.9 (7.1) | 9.3 (6.7) | .18 |
| Anxiety | 8.5 (6.9) | 7.4 (6.5) | .04 | 9.9 (7.4) | 8.9 (6.1) | .61 |
| Pre-trauma factors |  |  |  |  |  |  |
| Demographics |  |  |  |  |  |  |
| Gender |  |  |  |  |  |  |
| Female | 0.5 (0.5) | 0.5 (0.5) | .06 | 0.4 (0.5) | 0.5 (0.5) | .81 |
| Male | 0.5 (0.5) | 0.5 (0.5) |  | 0.6 (0.5) | 0.5 (0.5) |  |
| Age | 51.7 (18.5) | 53.5 (17.6) | .21 | 43.9 (15.3) | 48.0 (17.5) | .36 |
| Marital status |  |  |  |  |  |  |
| Never married | 0.2 (0.4) | 0.2 (0.4) | .12 | 0.4 (0.5) | 0.3 (0.5) | .69 |
| Married/cohabitating | 0.5 (0.5) | 0.5 (0.5) |  | 0.4 (0.5) | 0.3 (0.5) |  |
| Separated/divorced/widowed | 0.2 (0.4) | 0.2 (0.4) |  | 0.2 (0.4) | 0.3 (0.5) |  |
| Other | 0.0 (0.1) | 0.0 (0.1) |  | 0.0 (0.2) | 0.0 (0.0) |  |
| Missing | 0.0 (0.2) | 0.0 (0.1) |  | 0.0 (0.2) | 0.1 (0.2) |  |
| Education level |  |  |  |  |  |  |
| 0-11 years | 0.1 (0.3) | 0.0 (0.2) | .03 | 0.1 (0.3) | 0.1 (0.2) | .98 |
| 12 years (HS) | 0.3 (0.5) | 0.2 (0.4) |  | 0.2 (0.4) | 0.3 (0.5) |  |
| 13-15 years | 0.3 (0.5) | 0.3 (0.5) |  | 0.7 (0.5) | 0.6 (0.5) |  |
| 16+ years | 0.3 (0.5) | 0.4 (0.5) |  | 0.0 (0.2) | 0.1 (0.2) |  |
| (*continued*) | | | | | | |

| **Table S2. Attrition patterns for responses to traumatic stress and risk variables at the time of hospitalization (Time 1) by race/ethnicity.** | | | | | | |
| --- | --- | --- | --- | --- | --- | --- |
| **Variable at Wave 1** | **White (n = 654) all waves** | | | **Multiracial (n = 60) all waves** | | |
|  | No (n = 341) | Yes (n = 312) | *P* | No (n = 41) | Yes (n = 19) | *P* |
|  | mean (SD) | mean (SD) |  | mean (SD) | mean (SD) |  |
| Household income |  |  |  |  |  |  |
| Less than $35k | 0.3 (0.5) | 0.3 (0.5) | <.01 | 0.5 (0.5) | 0.7 (0.5) | .27 |
| $35K to less than $75k | 0.2 (0.4) | 0.2 (0.4) |  | 0.1 (0.3) | 0.1 (0.3) |  |
| $75K and more | 0.3 (0.4) | 0.3 (0.5) |  | 0.1 (0.3) | 0.2 (0.4) |  |
| Don't know/refused | 0.2 (0.4) | 0.1 (0.3) |  | 0.3 (0.4) | 0.1 (0.2) |  |
| Early life |  |  |  |  |  |  |
| Childhood home life | 3.5 (1.3) | 3.5 (1.3) | .47 | 2.7 (1.4) | 3.5 (1.4) | .04 |
| Caretaker dysfunction | 0.8 (1.1) | 0.6 (1.0) | .07 | 1.3 (1.4) | 0.9 (1.2) | .36 |
| Past trauma exposure |  |  |  |  |  |  |
| Lifetime sudden/terrible events ^a^ | 5.7 (8.4) | 4.8 (7.5) | .14 | 10.4 (14.8) | 6.6 (12.7) | .34 |
| Perceived discrimination |  |  |  |  |  |  |
| Everyday discrimination | 7.6 (8.2) | 6.7 (7.5) | .18 | 13.2 (11.8) | 7.8 (7.5) | .07 |
| Extreme discrimination | 1.5 (2.8) | 1.0 (2.1) | <.01 | 4.2 (4.5) | 1.6 (2.6) | .03 |
| Financial stress | 3.1 (2.2) | 2.8 (2.2) | .08 | 4.9 (2.2) | 4.0 (2.1) | .13 |
| Past mental health problems ^b^ | 1.2 (1.1) | 1.0 (1.0) | .02 | 1.5 (1.3) | 0.8 (0.8) | .03 |
| Peri-trauma factors |  |  |  |  |  |  |
| Subjective trauma severity ^c^ | 4.0 (2.1) | 4.1 (2.0) | .54 | 4.5 (2.0) | 3.2 (2.6) | .03 |
| Post-trauma factors |  |  |  |  |  |  |
| Expected social support | 22.9 (8.8) | 24.0 (8.3) | .09 | 20.0 (9.7) | 20.2 (9.7) | .94 |
| Social constraints | 4.0 (6.2) | 2.4 (4.6) | <.01 | 6.7 (7.8) | 3.3 (4.7) | .08 |
| *Notes:* | | | | | | |
| ^a^ Lifetime sudden/terrible events were winsorized to the 98th percentile to reduce the effect of extreme outliers. | | | | | | |
| ^b^ Measured through the question “In the past, how much of the time has feeling anxious, nervous, down, or depressed kept you from enjoying life?” | | | | | | |
| ^c^ Used two items about how “terrible” and “out of control” the event that brought participants to the hospital seemed. | | | | | | |

| **Figure S1. PTSD scores change over time in Latinx and White patients at varying levels of everyday discrimination, financial stress, past mental health stress, and social constraints at Time 1.** |
| --- |
| 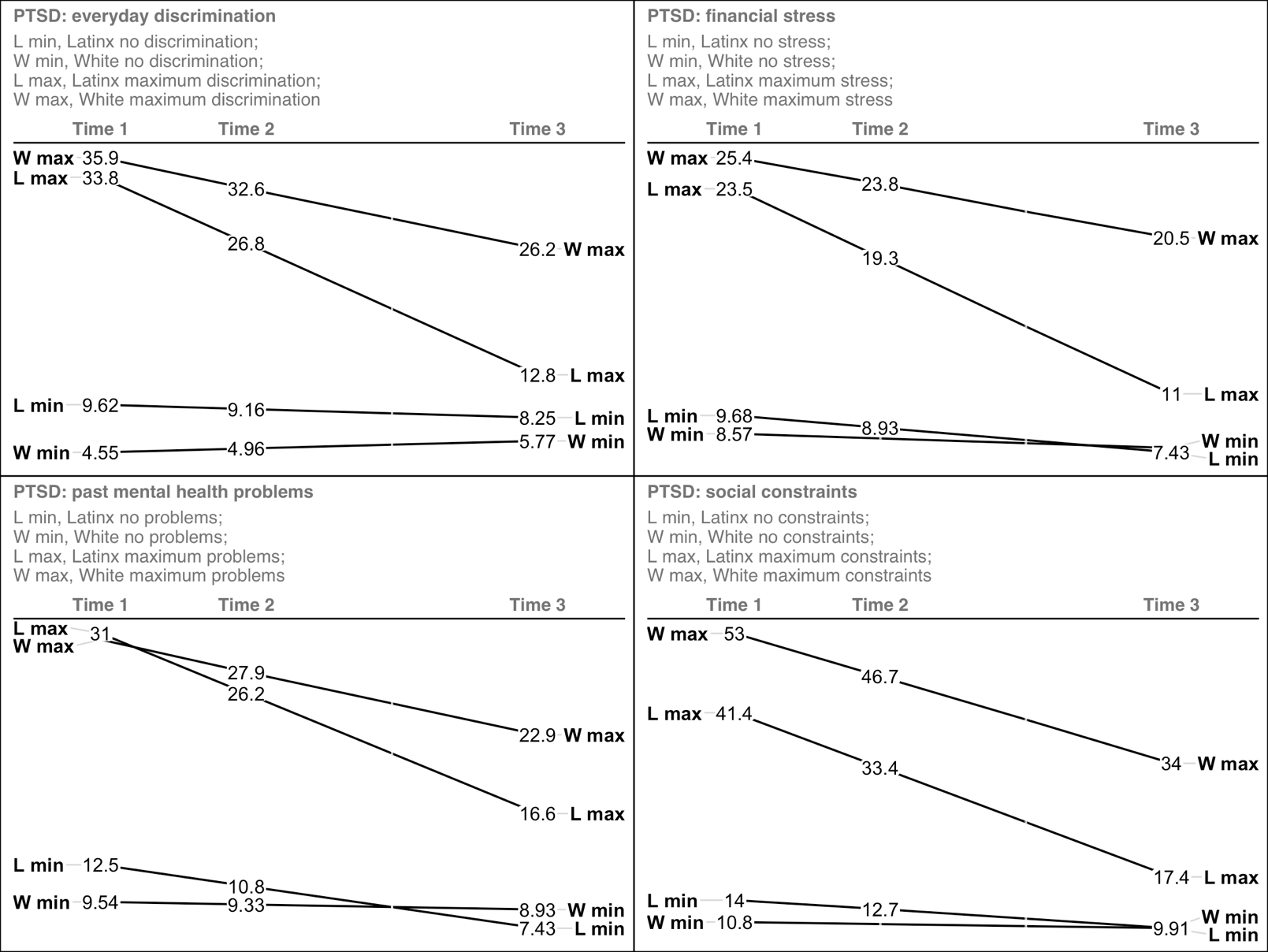 |

| **Figure S2. Depression scores change over time in Black and White patients at varying levels of everyday discrimination, financial stress, past mental health stress, and expected social support at Time 1.** |
| --- |
| 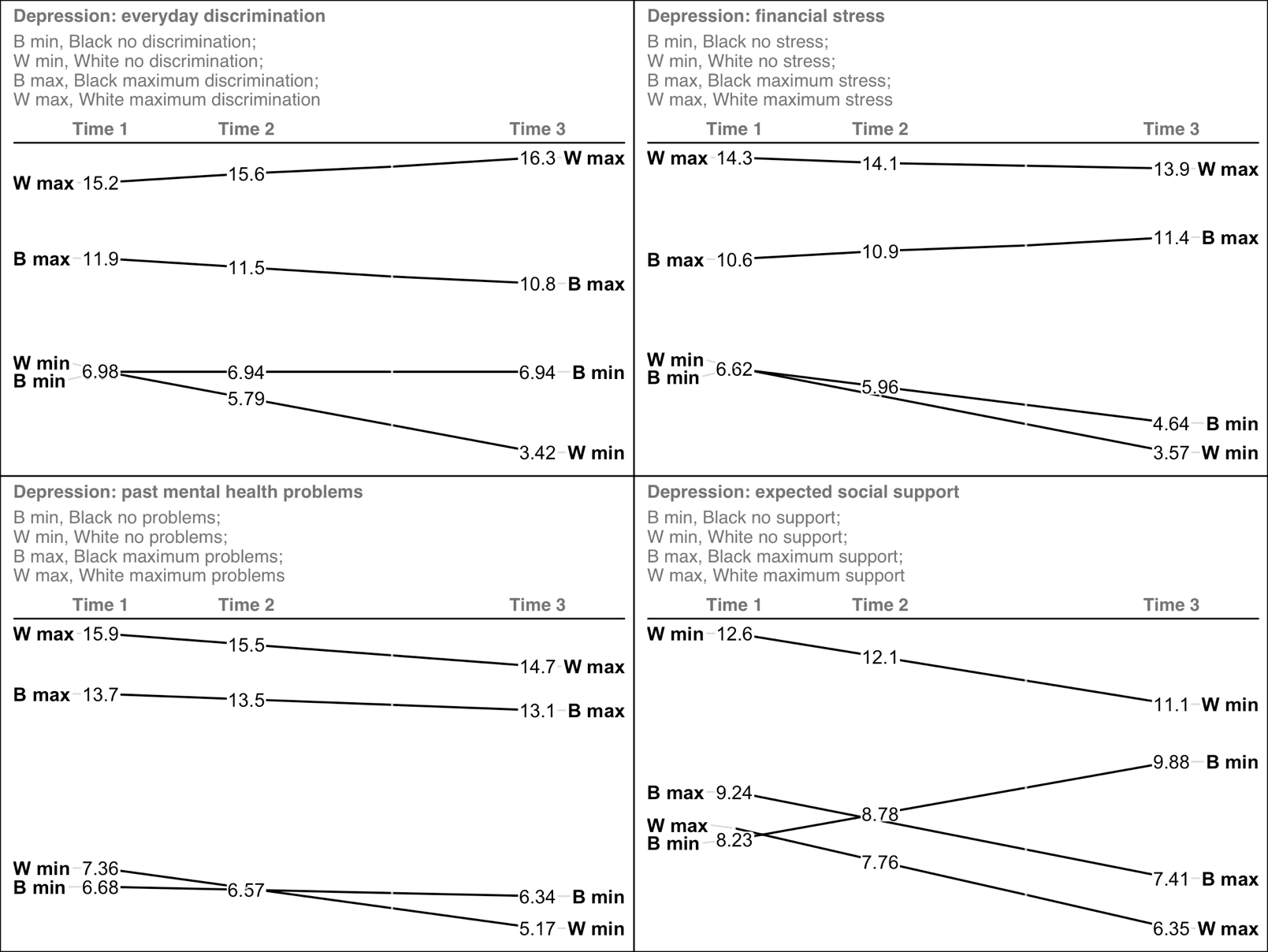 |

| **Figure S3. Anxiety scores change over time in Black and White patients at varying levels of financial stress, past mental health stress, expected social support, and social constraints at Time 1.** |
| --- |
| 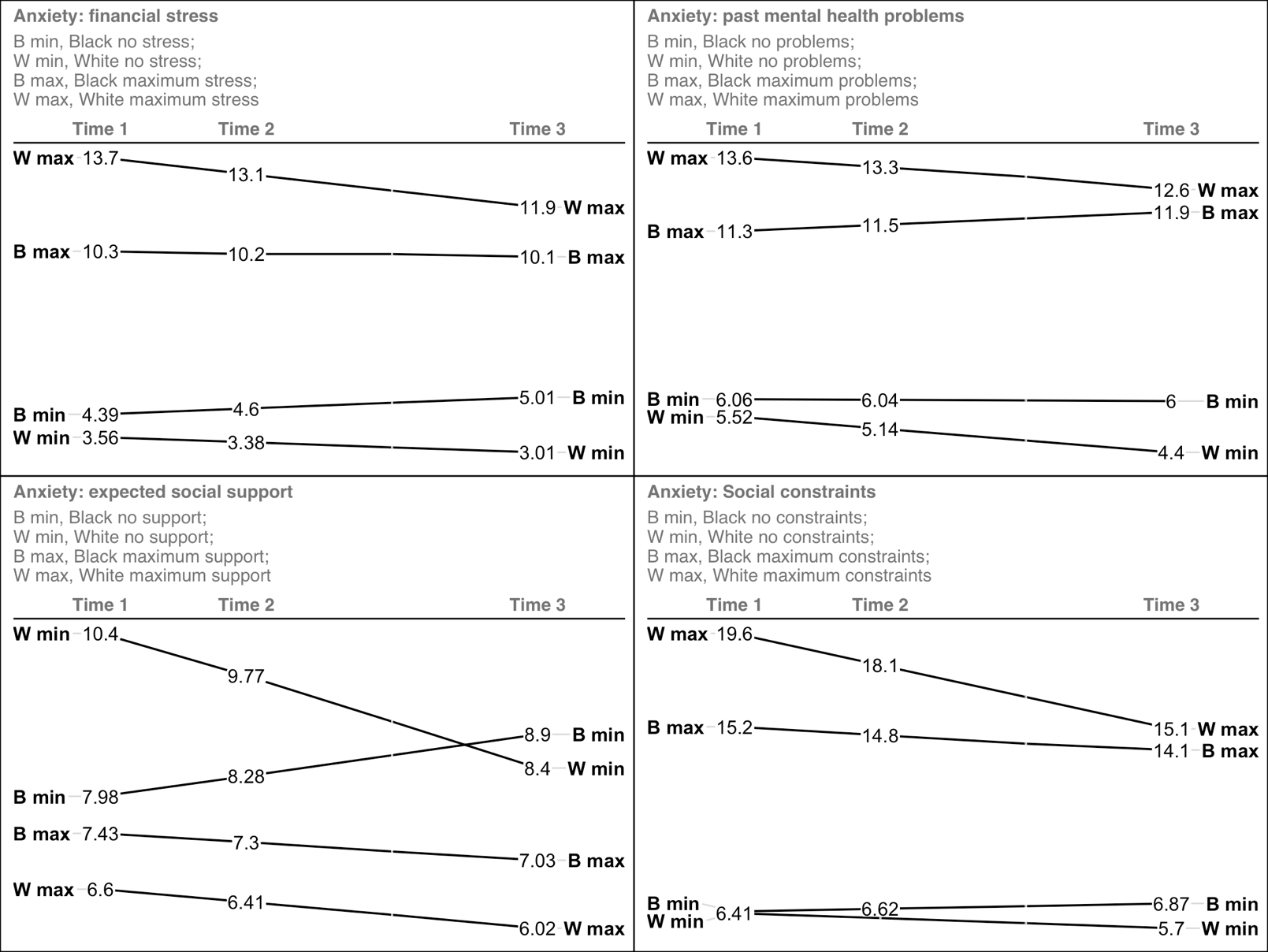 |
